# Insight Into the Molecular Dynamic Simulation Studies of Reactive Oxygen Species in Native Skin Membrane

**DOI:** 10.3389/fphar.2018.00644

**Published:** 2018-06-27

**Authors:** Dharmendra K. Yadav, Surendra Kumar, Eun-Ha Choi, Praveen Sharma, Sanjeev Misra, Mi-Hyun Kim

**Affiliations:** ^1^College of Pharmacy, Gachon University of Medicine and Science, Incheon, South Korea; ^2^Gachon Institute of Pharmaceutical Science & Department of Pharmacy, College of Pharmacy, Gachon University, Incheon, South Korea; ^3^Plasma Bioscience Research Center/PDP Research Center, Kwangwoon University, Seoul, South Korea; ^4^Department of Biochemistry, All India Institute of Medical Sciences, Jodhpur, India

**Keywords:** molecular dynamics simulation, reactive oxygen species, free energy profile, lipid peroxidation, oxidative stress

## Abstract

In recent years, the role of reactive oxygen species (ROS) in regulating cancer cell apoptosis, inflammation, cell ischemia, and cell signaling pathways has been well established. The most common sources of intracellular ROS are the mitochondrial electron transport system, NADH oxidase, and cytochrome P450. In this study, we investigated the dynamics and permeability of ROS using molecular dynamics (MD) simulations on native skin-lipid bilayer membranes. Native skin-lipid bilayers are composed of ceramide, cholesterol, and free fatty acid in an almost equal molar ratio (1:1:1). Dynamic distribution studies on ROS, i.e., hydrogen peroxide (H_2_O_2_) and O_2_ (^1^O_2_ by analogy), revealed that these species interact with cholesterol as a primary target in lipid peroxidation of the skin-lipid bilayer. Moreover, the permeability of ROS, i.e., H_2_O_2_, hydroxyl radicals (HO), hydroperoxy radical (HOO), and O_2_, along the skin-lipid bilayer was measured using free energy profiles (FEPs). The FEPs showed that in spite of high-energy barriers, ROS traveled through the membrane easily. Breaching the free energy barriers, these ROS permeated into the membrane, inflicting oxidative stress, and causing apoptosis. Collectively, the insight acquired from simulations may result in a better understanding of oxidative stress at the atomic level.

## Introduction

The stratum corneum, the outermost layer of the skin, is the primary barrier to many harmful molecules including reactive oxygen species (ROS) that penetrate the skin (Elias, 1983). Over the last few decades, the role of ROS has been documented in the process of aging and in several chronic diseases such as atherosclerosis, diabetes, Alzheimer’s disease, asthma, rheumatoid arthritis, and neurodegenerative diseases (Lacy et al., 1998; Bauerova and Bezek, 1999; Droge, 2002; Bahorun et al., 2006; Ivanova et al., 2013). ROS have also been implicated in many forms of cancer, such as melanoma (Fridman et al., 2007; Kim et al., 2009; Keidar et al., 2011; Arndt et al., 2013; Ishaq et al., 2014a), cervical (Leduc et al., 2009; Ahn et al., 2011), lung (Kim et al., 2011), breast (Kim et al., 2010; Wang et al., 2013), glioblastoma (Vandamme et al., 2012; Kaushik et al., 2013), and ovarian cancer (Iseki et al., 2012; Utsumi et al., 2013). ROS, i.e., superoxide (O^2−^) and hydroperoxy radical (HOO) are produced through the mitochondrial electron transport chain within cells. To address the toxicity of ROS, the host defense mechanism produces superoxide dismutase that converts O^2−^ into less reactive hydrogen peroxide (H_2_O_2_). However, in the presence of trace metals, H_2_O_2_ generates distinctly toxic hydroxyl radicals (HO) via fenton-type reactions (Cheng and Li, 2007; Prousek, 2007). Additionally, singlet oxygen, a prominent species in inducing melanoma, is produced via non-enzymatic oxidation of the skin-lipid bilayer at some point in the photosensitization reaction (Foote, 1968; Rawls and Van Santen, 1970). Consequently, these ROS makes the skin membrane vulnerable to peroxidative damage via triggering lipid peroxidation and targeting the sterol component of the skin-lipid bilayer (Logani and Davies, 1980; Girotti, 2008). Oxidized lipids present in the skin membrane have the ability to modify the membrane properties, especially in terms of permeability (Grzelińska et al., 1979; Richter, 1987; Wratten et al., 1992; Chen and Yu, 1994; Tai et al., 2010; Jurkiewicz et al., 2012). Furthermore, the distribution of various types of ROS at the interface between the lipid bilayer and water facilitates the understanding of lipid peroxidation and radical scavenging via membrane antioxidants in an organism (Afri et al., 2002; Gamliel et al., 2008). In recent years, cold atmospheric plasmas (CAPs) have been proven to have great potential in different areas of medical science, including melanoma research. The possible selectivity toward melanoma might be due to the substantial rise in intracellular ROS in response to oxidative stress occurring in membranes of cancer cells compared to that in normal cell membranes upon the same CAP treatment (Ja Kim et al., 2013; Ishaq et al., 2014a,b; Kaushik et al., 2014; Kim and Chung, 2016). However, the underlying mechanisms for the enhanced concentration of ROS in cancer cell membranes remain elusive (Van der Paal et al., 2016). Another explanation could be the increased expression of aquaporins (AQPs), a membrane protein family that facilitates the diffusion of water across cellular membranes, in melanoma/non-melanoma cells (Verkman et al., 2008; Ramadan et al., 2017). It is shown that various cancer cell lines have expressed elevated levels of certain AQPs (Shi et al., 2012; Papadopoulos and Saadoun, 2015; Yan et al., 2015, 2017). Moreover, because of the similarity between ROS (i.e., H_2_O_2_, HOO, HO, O_2_) and water (Bienert et al., 2006; Bienert and Chaumont, 2014), AQPs are able to facilitate the passive diffusion of these ROS through membranes, which leads to increased oxidative stress.

The skin-lipid bilayer membrane is the outermost cellular organ that acts as a barrier and controls the transportation of materials. Several experimental studies on phospholipid vesicles as simple models for cell membranes have been accomplished to infer the oxidation of lipids, structural and chemical modifications, and transportation of ROS through the phospholipid membranes (Hammer et al., 2013; Hong et al., 2014; Tero et al., 2014; Szili et al., 2015; Ki et al., 2016; Maheux et al., 2016). In addition, once the lipids are oxidized, they accumulate within the bilayer and further contribute to changes in structural organization and packing of membrane lipid components. Consequently, those structural changes abate the mechanical strength, permeability, and fluidity of the lipid bilayer membrane (Beranova et al., 2010; Jarerattanachat et al., 2013; Van der Paal et al., 2016).

The present work describes how the native skin-lipid bilayer was constructed and simulated. Subsequently, we performed the dynamics and distribution of ROS (especially H_2_O_2_ and O_2_) at the interface between the skin-lipid bilayer and water, and throughout the lipid bilayer. Since H_2_O_2_ is the primary species that undergoes fenton-type reaction to generate various radicals and O_2_, as an analogy of ^1^O_2_, has a prime role in lipid oxidation, particularly in the sterol component of the skin-lipid bilayer (Engelmann et al., 2007; Cordeiro et al., 2012). Thus, we further investigated the interaction of ROS (H_2_O_2_ and O_2_) with hydrophilic headgroups of the skin-lipid bilayer. Furthermore, the permeability of different ROS, i.e., H_2_O_2_, HO, HOO, and O_2_ (^1^O_2_ by analogy), through the skin-lipid bilayer membrane was evaluated using the umbrella sampling (US) method by calculating the potential mean of forces (PMFs) (Roux, 1995; Van Der Spoel et al., 2005). Since these ROS have different tendencies for permeation, thus from the PMFs, the free energy barriers for permeation across the lipid bilayer may be derived. The sampling of the entire system can be advantageous in the US method as compared to the traditional molecular dynamics (MD) simulation. Indeed, Cordeiro (2014) used the US method to calculate the FEPs of various ROS in POPC (1-palmitoyl-2-oleoyl-sn-glycero-3-phosphocholine) membranes and observed at par with experimental outcomes. Similarly, Gajula et al. (2017) carried out multi-scale simulation studies on the permeation of various solutes through the skin-lipid bilayer membrane. The present study extends the US method to study the permeation of ROS through the skin-lipid bilayer membrane.

## Materials and Methods

### Computational Scheme

**FIGURE 1:**
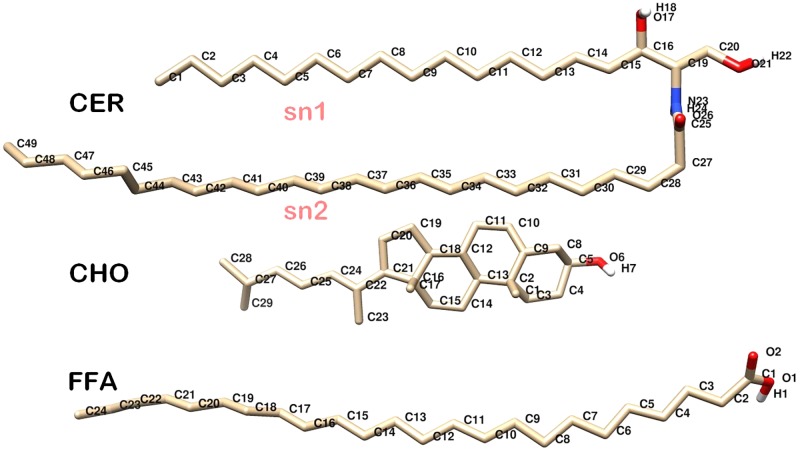
Molecular structure of individual skin lipids used in simulations. CER, Ceramide NS; CHO, Cholesterol; FFA, Free Fatty Acid. Oxygen, Hydrogen, Carbon, and Nitrogen atoms are shown in red, white, gray, and blue color, respectively.

The structures of the individual components of native skin-lipid bilayer used in this study, which are ceramide (CER), cholesterol (CHO), and free fatty acid (FFA), are shown in **Figure 1**. The skin-lipid bilayer was constructed using the PACKMOL package (Martinez et al., 2009). The native skin-lipid bilayer includes 52 CER, 50 CHO, and 52 FFA at an almost equal molar ratio (1:1:1) and 5120 water molecules. The force field parameters for CER, CHO and FFA were based on prior studies (Berger et al., 1997; Höltje et al., 2001) and proved to be steady with experimental results. The CH_2_ and CH_3_ groups of CER were represented by united carbon atoms with zero partial charge, and the polar hydrogen atoms were included explicitly (Das et al., 2009). The simple point charge model was used for the water molecules (Berendsen et al., 1981). The ROS parameters were taken from a study reported by Cordeiro (2014; Supplementary Table S1).

All simulations were performed in the constant-temperature, constant-pressure ensemble (NPT) using the GROMACS 5.1.4 MD package (Berendsen et al., 1995; Van Der Spoel et al., 2005; Hess et al., 2008; Pronk et al., 2013). The temperature was controlled at 310.15 K by a Nose–Hoover thermostat with a time constant of 0.5 ps and coupled separately to lipid or water molecules. Pressure was controlled at 1 bar using a Parrinello–Rahman barostat with a time constant of 5 ps and compressibility of 4.5 × 10^−5^ bar with semi-isotropic coupling. A time step of 2fs was used for all simulations. The cut-off distances for columbic interactions and van der Waals interactions were both set at 1.2 nm. The system was periodic in all Cartesian directions.

**FIGURE 2:**
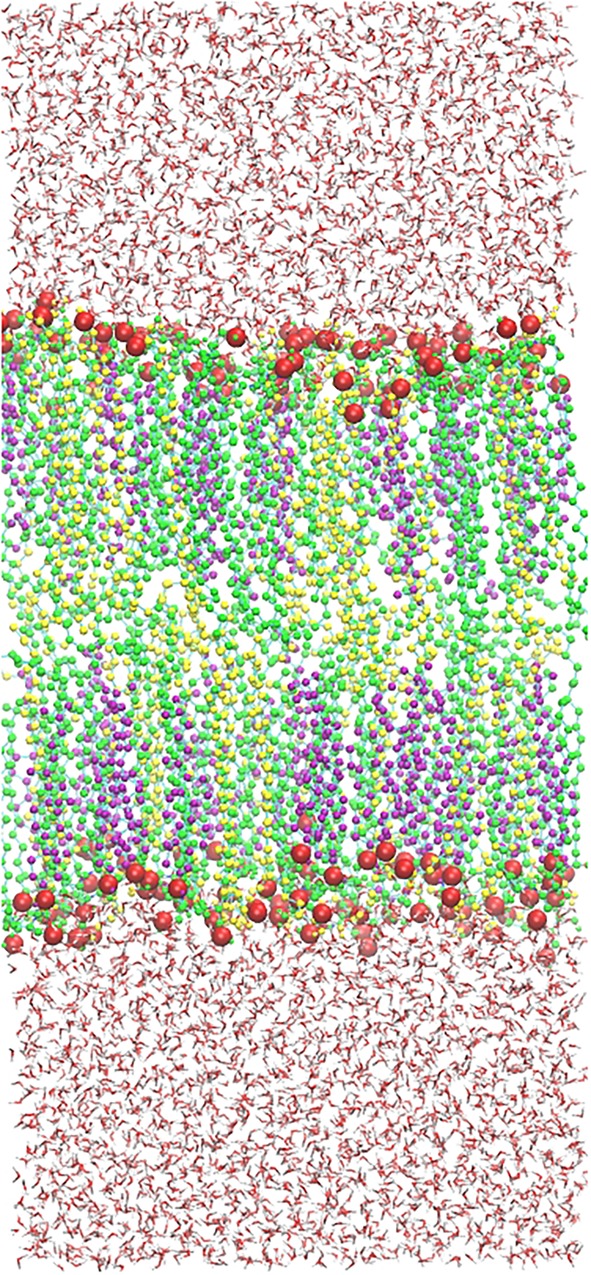
Native structure of a skin lipid bilayer containing 52 CER (Green), 50 CHO (Purple), 52 FFA (Yellow) molecules, and 5210 water molecules. Polar heads (Oxygen) are shown in red sphere [van der Waals (VDW)].

The built skin-lipid bilayer was first energy-minimized using the steepest descent algorithm, followed by constant-temperature, constant-volume ensemble (NVT) equilibration for 2 ns under restrained conditions. The equilibrated bilayer was further simulated for 10 ns under NPT ensemble before the structure was submitted to simulate annealing, where the system was heated upto 360 K and cooled to 310.15 K in a systematic manner to obtain well-hydrated lipid bilayer heads. The system was further equilibrated for 50 ns, followed by 200 ns of production simulation under NPT ensemble conditions. The size of the obtained bilayer was 5.02 nm × 5.02 nm × 11.3 nm. The final structure of the native skin-lipid bilayer structure (**Figure 2**) was used to study the membrane properties, i.e., area per lipid (APL), bilayer thickness, and tail order parameters. Furthermore, the modeled native skin-lipid bilayer structure was used to study the ROS distribution at the interface between the lipid membrane and water, as well as to estimate the free energy profiles (FEPs).

### Analysis of Native Skin-Lipid Bilayer Membrane Properties

The membrane properties, i.e., APL, bilayer thickness, and tail order parameters, of CER and FFA were measured from the simulated native skin-lipid bilayer membrane. The APL describes the packing of a lipid bilayer. To calculate APL, the box-x vector of the lipid bilayer was defined along with estimates of error on last 50 ns of the simulation. Similarly, the membrane thickness, an important parameter in describing the properties of the system, was calculated using the APLVORO software (Lukat et al., 2013) on last 50 ns of the simulation trajectory. In this analysis, the headgroups of each lipid component (i.e., atoms O21 for CER, O6 for CHO, and O_2_ for FFA) were used as the key atoms, and membrane thickness was defined as the average position between the headgroups of both leaflets. Finally, the ordering or alignment of the lipid hydrocarbon tails of CER and FFA could be determined by defining the tail order parameters (*S*_z_) per carbon atom *C*_n_, as follows:

$$S_{\text{z}}=\frac{3}{2}\langle \cos^{2}(\theta_{\text{z}})\rangle -\frac{1}{2}$$

where *𝜃*_z_ is the angle between the vector from *C*_n−1_ to *C*_n+1_ and the bilayer normal (*z*-axis). Here *S*_z_ = 1 represents perfect alignment of the tails with the *z*-axis, *S*_z_ = 0 represents random orientation, and *S*_z_ = −0.5 represents perpendicular alignment to the bilayer normal (Gupta and Rai, 2015; Piggot et al., 2017).

### Simulations of ROS (H_2_O_2_ and O_2_) Distribution and Dynamics

Hydrogen peroxide is a primary ROS that is produced by mitochondria in events mediated by external stimuli (i.e., exposure of cells to UV or other oxidative stress conditions). It plays a role in apoptotic signaling via cell-to-cell transmission (Rojkind et al., 2002; Pletjushkina et al., 2006). In the event of fenton-type reaction, H_2_O_2_ generates toxic radicals, i.e., HO (Prousek, 2007). In addition, singlet oxygen (^1^O_2_) species play an important role in lipid oxidation via photosensitization reactions, targeting the sterol component of the lipid bilayer and similarly disrupting the integrity and fluidity of the lipid bilayer. Thus, in order to comprehend the interactions and distributions of ROS, especially H_2_O_2_ (as a primary species) and O_2_ (^1^O_2_ by analogy), the lower (25 molecules) and higher (50 molecules) concentrations of each ROS were placed separately over lipid bilayer head in two pre-equilibrated system. The concentrations selected corresponds to those found in mitochondria (experimentally measured molar fraction of ca. 0.5%) (Cadenas and Davies, 2000). After 20 ns of restrained equilibration under NPT ensemble, each system was simulated for 30 ns at 310.15 K and 1 bar pressure. The density profile along the *z*-direction and the distance between the ROS (H_2_O_2_ and O_2_) and the upper headgroups of the skin-lipid bilayer membrane were evaluated.

### Permeation Free Energy Profiles

**FIGURE 3:**
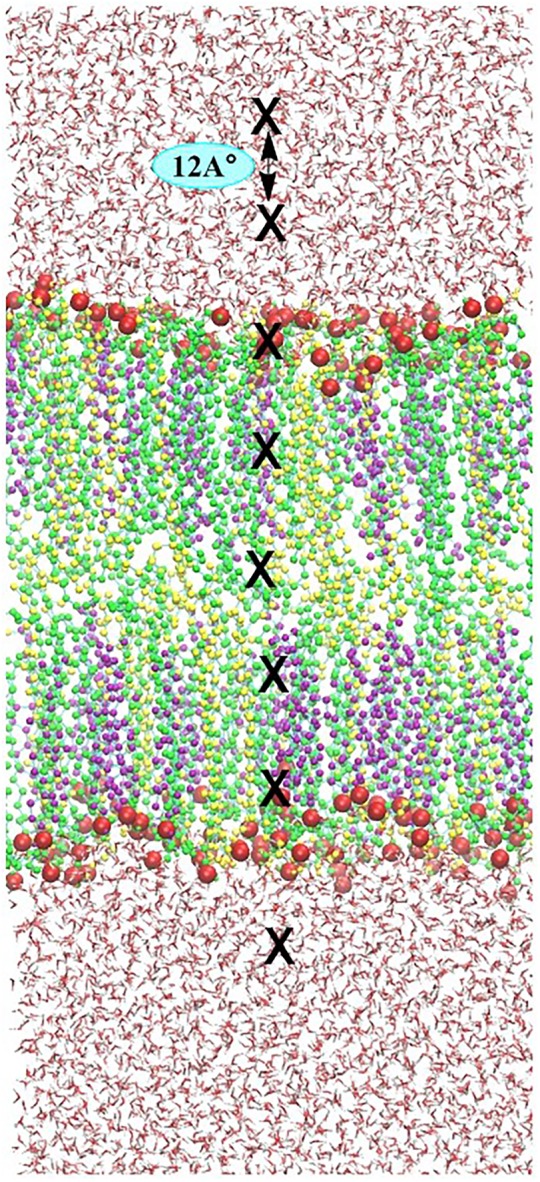
Illustration of the umbrella sampling (US) simulation set-up. Eight umbrella windows separated by 12 Å (position of ROS depicted by block crosses) are sampled in one simulation. In consecutive simulations, each species is shifted by 0.12 Å. To sample the entire membrane system, 33 simulations are performed, yielding a total of 264 umbrella histograms from which a potential mean of forces (PMF) is constructed.

The FEPs of each ROS molecule throughout the skin-lipid bilayer was calculated by the US methodology. The US methodology makes use of additional energy terms, known as bias, in the system to ensure efficient sampling throughout the reaction coordinate. The US method has already been described in the literature (Gupta et al., 2016; Gajula et al., 2017; Van der Paal et al., 2017), however, in brief, for sampling the entire phase of the skin-lipid bilayer membrane. Starting from the upper water leaflet, crossing the lipid bilayer structure, and ending in the lower water leaflet, multiple ROS molecules were placed in the native skin membrane system at different *xy*- and *z*-planes. The reaction coordinate of the system was chosen in the *z*-dimension, where *z* = 0 corresponds to the center of mass (COM) of lipid molecules (CER + CHO + FFA). To save computational resources, eight umbrella windows were sampled during each simulation, keeping a distance of 1.2 nm (12 Å) among consecutive windows, starting at ∼4.8 nm from the COM of the bilayer as shown in **Figure 3**. For each ROS, multiple systems were created. Each system was energy-minimized and equilibrated under NPT ensemble, while keeping the ROS molecules fixed at the current position. Each US simulation lasted for 20 ns, and last 10 ns were used for analysis, i.e., to acquire the US histograms and to calculate the FEPs. In each US simulation, the ROS molecules were free to move in the *xy*-plane, but their movement in the *z*-direction was constrained by applying a harmonic bias with a spring constant of 2000 kJ⋅mol^−1^⋅nm^−2^. FEPs are constructed via the weighted histogram analysis method (WHAM), as implemented in GROMACS (Hub et al., 2010). To improve the statistical accuracy of sampling, the final energy profiles were obtained by averaging six FEPs for each ROS.

## Results and Discussion

### Calculation of Native Skin-Lipid Bilayer Membrane Properties

The APL of native skin-lipid bilayer is shown in Supplementary Figure S1. The calculated APL was 0.32 ± 0.003 nm, which was comparable to that reported in previous studies (0.31 nm) (Gupta and Rai, 2015) and (0.33 ± 0.01 nm) (Wang and Klauda, 2018). Similarly, the thickness of the native skin-lipid bilayer membrane was calculated on the last 50 ns of simulation and was found to be 4.98 nm, which was comparable to those reported in previous experiments (5.12 nm) (Gupta and Rai, 2015) and (5.17 nm) (Das et al., 2009).

Likewise, the tail order parameters were calculated for the hydrophobic chains of CER and FFA, and are shown in Supplementary Figure S2. The order parameters of CER for sn1 and sn2 are low near C16 and C24, respectively, and increased as we moved toward the mid-bilayer and decreased further down toward the center of the bilayer. Moreover, in the presence of CER and CHOL, the order parameters for FFA chain length followed the similar trend as that for the CER chain. Similar chain lengths of CER and FFA led to proper inter-digitization and ordering of tails.

### Distribution and Dynamics of ROS at the Membrane and Water Interface

**FIGURE 4:**
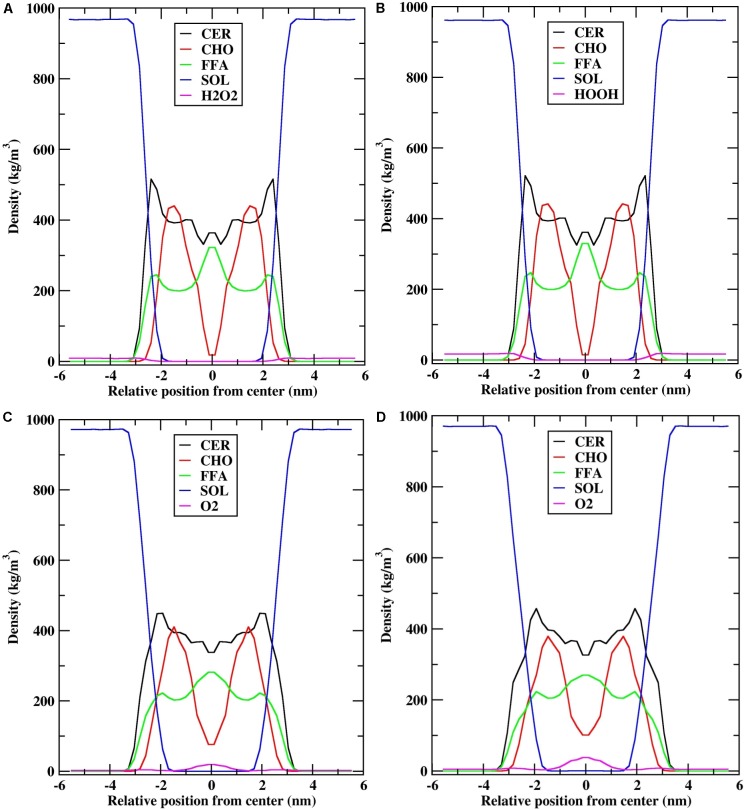
Density profile of individual lipid components (CER-CHO-FFA) along the bilayer normal (*z*). **(A)** H_2_O_2_-25; **(B)** H_2_O_2_-50; **(C)** O_2_-25; and **(D)** O_2_-50.

To understand the arrangement of the different components in each system under investigation, the density profiles of the individual lipid components and ROS along the *z*-direction were plotted, which are shown in **Figure 4**.

The density distribution for the individual lipid components and H_2_O_2_ species (H_2_O_2_-25: 25 molecules of H_2_O_2_, H_2_O_2_-50: 50 molecules of H_2_O_2_) were qualitatively similar (**Figures 4A,B**). The density profile of CER showed high density near the headgroups region, low density at the bilayer center, and almost constant density between these two bilayer regions, which was due to the ordered tail atoms. The density profile of FFA is similar to that of CER, except that high density was observed at the bilayer center. Moreover, the density profile of CHO showed two shoulders at *z* = ∼1.5 nm and low density at the bilayer center, suggesting that because of the shorter chain length of CHO, they mostly resided at the interface between the lipid membrane and water, and the alkyl tails were aligned with the alkyl chains of CER. Furthermore, because of the shorter chain length of CHO, the bilayer center consists primarily of CER and FFA tails and they overlapped with each other. The distribution of H_2_O_2_ was mostly at the interface between the lipid membrane and water, and no H_2_O_2_ molecule was found inside the bilayer center (Supplementary Figure S3).

Likewise, the density distributions of O_2_-25 (25 molecules of O_2_) and O_2_-50 (50 molecules of O_2_) were qualitatively similar to each other. However, they were different from the density profiles of H_2_O_2_ (**Figures 4C,D**). During the simulation, the distribution of the O_2_ species affected the density profile of all lipid components. CER had a shoulder in the headgroups region, while the height of the shoulder decreased. The densities of CHO and FFA at the bilayer center decreased, corresponding to the fact that the bilayer center was occupied by O_2_ molecules (Supplementary Figure S4). In addition, the density of O_2_ was higher at the bilayer center in O_2_-50, whereas it was slightly lower in O_2_-25, suggesting that as the number of O_2_ molecules increased, they penetrated deeper into the bilayer and occupied the space between the two leaflets. However, the lipid bilayer membrane preserved its symmetry with little perturbation.

**FIGURE 5:**
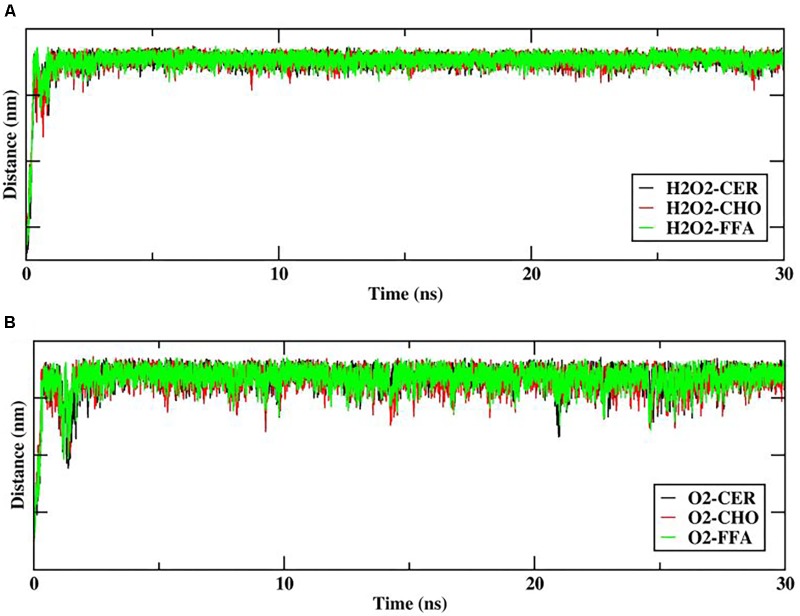
Distance between reactive oxygen species (ROS) **(A)** hydrogen peroxide (H_2_O_2_), **(B)** O_2,_ and headgroups of upper skin-lipid bilayer components (CER-CHO-FFA) as a function of time (ns).

In order to explore the interactions/contacts of H_2_O_2_ and O_2_ species with the lipid bilayer membrane components, the distances between the H_2_O_2_ or O_2_ species and the headgroups of the upper lipid bilayer were measured and are shown in **Figure 5**.

**FIGURE 6:**
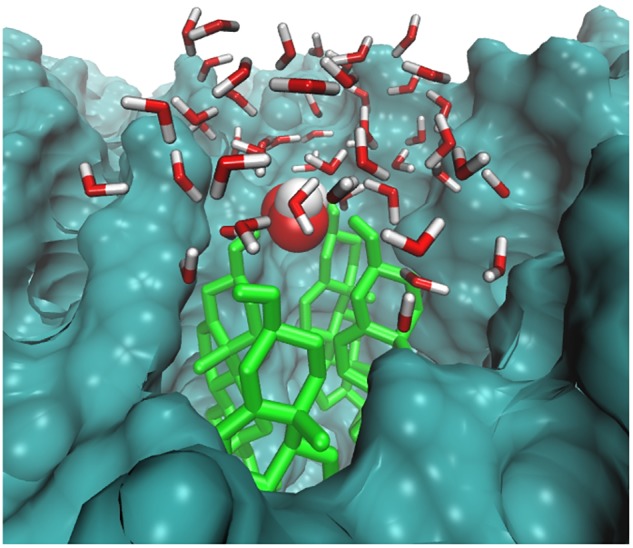
Simulation snapshot showing H_2_O_2_ species in the headgroups regions at 20.6593 ns. The H_2_O_2_ species is presented according to element color. The cholesterol is shown in light green color. The rest of the bilayer is shown as surface representation with dark green color. The water molecules within 8 Å are shown with the default color.

The distance between H_2_O_2_ and the headgroups of the upper lipid bilayer is shown in **Figure 5A**. Furthermore, H_2_O_2_ as primary species may generate various species that mainly targets hydrophilic or double-bond containing lipid components. Thus as the simulation progressed, all H_2_O_2_ species made multiple contacts with the headgroup of the lipid component. **Figure 5A** clearly shows that among all of the headgroups, H_2_O_2_ made multiple contacts with the headgroups of CHO. Moreover, selected snapshots showed the interaction profile between H_2_O_2_ and CHO, in which H_2_O_2_ was surrounded by the headgroups of CHO molecules (**Figure 6** and Supplementary Figure S3), revealing that in *in situ* fenton-type reactions, H_2_O_2_ may generate other species that structurally modify CHO to cause perturbational changes in the skin-lipid bilayer structure that may result in oxidative stress.

**FIGURE 7:**
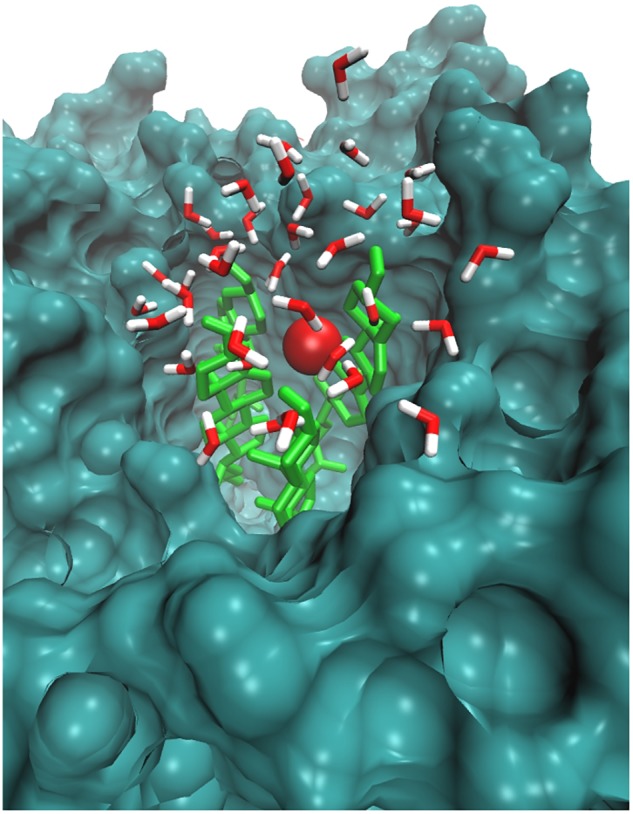
Simulation snapshot showing O_2_ species in the headgroups regions at 25.4265 ns. The O_2_ species is presented according to element color. The cholesterol is shown in light green color. The rest of the bilayer is shown as surface representation with dark green color. The water molecules within 8 Å are shown with the default color.

Likewise, Cordeiro (2014) simulated the O_2_ species in POPC lipid bilayers and stated that O_2_ prefers to reside within the interior membrane. Since the role of singlet oxygen in sterol peroxidation has already been established (Kulig and Smith, 1973), we further investigated its role in interactions with native skin-lipid bilayers. The distance between the O_2_ species and the headgroups of upper lipid bilayers was measured, which is shown in **Figure 5B**. During simulation, the O_2_ species made maximum multiple contacts with the headgroups of CHO in the upper skin-lipid bilayer. To show the interaction profile between O_2_ and the CHO headgroups, a snapshot was selected as a representative (**Figure 7** and Supplementary Figure S4). The interaction profile and final simulated structure revealed that besides preferring to reside in the interior of the bilayer, some O_2_ species also engaged with the CHO headgroups. The final simulated structure showed that during simulation, O_2_ traveled from the upper aqueous layer through the lipid bilayer toward the bilayer center. This was supported by comparing the distance between H_2_O_2_ or O_2_ and the headgroups of the upper skin-lipid bilayer, and a maximum distance for O_2_ as compared to H_2_O_2_ was observed. These findings supported the fact that while traveling alongside the skin-lipid bilayer, O_2_ might have had access to potential peroxidation sites (cholesterol is more prone to undergo peroxidation that can result in perturbational changes in the membrane (Neto and Cordeiro, 2016).

### Effect of ROS on Skin-Lipid Bilayer Membrane Permeability

**FIGURE 8:**
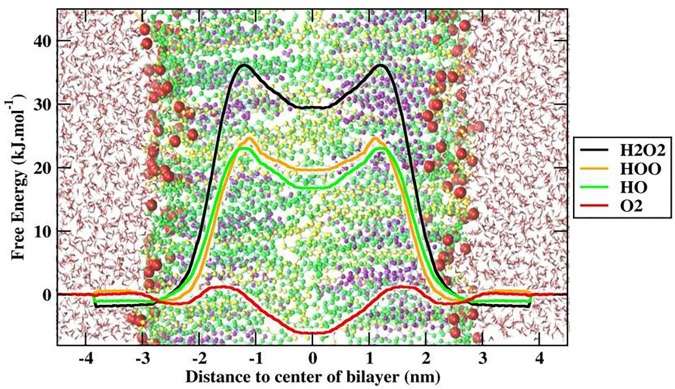
Free energy profile (FEP) of different ROS across skin-lipid bilayer (CER-CHO-FFA) membrane.

To better explain and understand the permeability of various ROS through the skin-lipid bilayer membrane, averaged PMF profiles were measured for all ROS and are shown in **Figure 8**. The bootstrapping standard deviations from PMF profiles are shown in Supplementary Figure S5. In **Figure 8**, the membrane shown on the background depicts the location of different regions of the bilayer structure, and the transfer free energy barriers associated with these profiles are compiled in **Table 1**. In **Figure 8**, H_2_O_2_, HO, and hydroperoxyl radical (HOO) displayed energy minima before entering the lipid bilayer near the headgroups region (∼−2.9 nm), which corresponded to interactions with the headgroups of the lipid bilayer. However, because of the additional oxygen atom, the HOO species displayed greater interaction compared to HO. Meanwhile, the O_2_ species showed a slight increase in free energy (∼0.25 kJ/mol) near the headgroup region. These observations are consistent with those presented in a previous study by Gupta et al. (2016), where the free energy of hydrophilic species slightly decreased but that of hydrophobic species slightly increased. Moving toward the bilayer center, the role of lipid bilayer membrane as a permeation barrier can be explained from the drastic increase in free energy with all ROS species except for O_2_. The transfer free energy increased and reached a maximum in the hydrophobic core of H_2_O_2_ (∼37.61 kJ/mol), HOO (∼25.04 kJ/mol), and HO (∼24.06 kJ/mol), and then decreased to ∼30.51, ∼20.20, and ∼16.62 kJ/mol, respectively, at the bilayer center. However, because of the hydrophobic nature of the O_2_ species, its PMF profile was entirely different from those of other types of ROS. The transfer free energy after crossing the headgroup region of the lipid bilayer decreased to ∼-1.32 kJ/mol and then increased to ∼2.66 kJ/mol. This new energy barrier at ∼1.5 nm for O_2_ can be assigned to the bulky ring of cholesterol in the skin-lipid bilayer. The transfer free energy further decreased (∼-6.1 kJ/mol) and displayed an energy-minimum at the bilayer center because of the depletion of lipids in the center and contributed to the accumulation of O_2_ species at the center of the skin-lipid bilayer (Subczynski and Hyde, 1983). Comparing the free energy barriers of all ROS, the permeation of H_2_O_2_ was most hindered with an activation free energy estimated to be 37.61 ± 1.92 kJ/mol, which is comparatively higher than other computational (33 ± 4 kJ/mol) (Cordeiro, 2014) and experimental (36.8 kJ/mol) (Mathai and Sitaramam, 1994) findings. Such differences in transfer free energy may be due to the high ordering of CER and FFA chains and the existence of the native skin-lipid bilayer in the gel phase (Paloncyova et al., 2014; Gupta and Rai, 2015). The free energy barriers for HO and HOO are highly similar and there is almost no significant barrier for O_2_, which is in agreement with the preceding experimental study and corresponds to the permeability of different types of ROS (Simon et al., 2008). All PMF profiles showed local energy minima in the center of the bilayer that correspond to regions with decreased lipid bilayer density. **Figure 4** displays the density profile of the lipid components, and the decrease in the density of cholesterol in the center of the bilayer can be attributed to the smaller structure of cholesterol. Moreover, the notably higher free energy barriers for H_2_O_2_, HO, and HOO can be explained from the observation that these species established multiple hydrogen bonding with the surrounding water molecules, and as soon as they lose their hydration water before penetrates into the lipid membrane. In fact, Cordeiro (2014) showed that H_2_O_2_ can build up twice the number of H-bonds (4.5) compared with HO (2) and HOO (2.3).

**Table 1:** Transfer free energies (ΔG) of all investigated reactive oxygen species (ROS) in a native skin-lipid bilayer (CER-CHO-FFA) membrane.

ROS	ΔG (kJ.mol^−1^)
H_2_O_2_	37.61 ± 1.92
HOO	25.03 ± 1.75
HO	24.06 ± 1.07
O_2_	−4.70 ± 0.57

## Conclusion

Hydrogen peroxide is the primary ROS produced in mitochondria in events caused by oxidative stress. H_2_O_2_ further undergoes fenton-type reactions *in situ* to generate various radicals/species. Along with these species, singlet oxygen (^1^O_2_) has been shown to hamper the fluidity and integrity of skin-lipid bilayer membranes by peroxidizing sterol, one of the key components of skin-lipid bilayer membranes. The present study investigated the interaction of H_2_O_2_ and O_2_ (^1^O_2_ by analogy) with the headgroups of skin-lipid bilayer membranes and estimated the permeability of various types of ROS, i.e., H_2_O_2_, HO, HOO, and O_2_, across the skin-lipid bilayer membrane. The results obtained in the present study illustrated that H_2_O_2_ and O_2_ make multiple contacts with the headgroups of the skin-lipid bilayer membrane, and among the different headgroups, cholesterol has maximum contact with these species. The study also showed that besides residing on the interface between the skin-lipid bilayer and water, O_2_ also prefers to concentrate more in the center of the skin-lipid bilayer by traveling along the lipid tail of the skin-lipid bilayer membrane. Thus, whilst O_2_ travels along the lipid bilayer, it might have gained access to potential peroxidation sites of the lipid components. The major components of skin-lipid bilayers are ceramide, cholesterol, and free fatty acid, and peroxidation studies have already proven that cholesterol is more prone to peroxidation *in situ*. Thus, the present study supported the experimental evidence. We also determined the permeability of various types of ROS across the skin-lipid bilayer membrane. The FEPs in terms of PMFs indicated that in spite of the high free energy barrier, ROS (i.e., H_2_O_2_, HO, and HOO) in the native skin-lipid bilayer membrane easily travel across the membrane. The minimal free energy barrier of O_2_ suggests that these species do not have any barrier. Thus, they are easily transported along the skin-lipid bilayer, and reside in the bilayer center. By breaching the free energy barriers, all ROS would be able to permeate the membrane, causing oxidative stress that might lead to apoptosis. All of the results were obtained for the native skin-lipid bilayer membrane (equimolar ratio of lipid components). However, further investigation will be performed with varying concentrations of cholesterol, as it is the primary target for lipid peroxidation. Collectively, the insights obtained from simulations might help in gaining a better understanding of oxidative stress at the atomic level.

## Author Contributions

DY conceived and designed the project and collected data from the literature and databases. SK and DY performed the experiments, analyzed the data, and wrote the manuscript. M-HK, E-HC, PS, and SM provided the molecular modeling lab facility. All authors contributed to the interpretation and discussion of the results and read and approved the final version of the manuscript.

## Conflict of Interest Statement

The authors declare that the research was conducted in the absence of any commercial or financial relationships that could be construed as a potential conflict of interest.
