# Supplementary material for: Insight Into the Molecular Dynamic Simulation Studies of Reactive Oxygen Species in Native Skin Membrane
Source: Front Pharmacol. 2018 Jun 27;9:644. doi: 10.3389/fphar.2018.00644 (PMC6030362; doi:10.3389/fphar.2018.00644)
Supplement: Supplementary file 1 [file Data_Sheet_1.DOC]

**SUPPLEMENTARY FILE**

**Insight into the Molecular Dynamic Simulation Studies of Reactive Oxygen Species in Native Skin Membrane**

Dharmendra Kumar Yadav^a,$*^, Surendra Kumar^a,$^, Eun-Ha Choi^b^, Praveen Sharma^c^, Sanjeev Misra^c^, Mi-Hyun Kim^a,*^

^a^College of Pharmacy, Gachon University of Medicine and Science, Hambakmoeiro, 191, Yeonsu-gu, Incheon 406-799, Korea

^b^Plasma Bioscience Research Center/PDP Research Center, Kwangwoon University, Nowon-Gu, Seoul 139-791, Korea

^C^Department of Biochemistry, All India Institute of Medical Science, Jodhpur, Rajasthan, 342005, India

*Email:* [*dharmendra30oct@gmail.com*](mailto:dharmendra30oct@gmail.com)*, k*[*mh0515@gachon.ac.kr*](mailto:mh0515@gachon.ac.kr)

*corresponding author

**Dr. Dharmendra Kumar Yadav, Ph.D**

Research Assistant Professor

Office: +82-32-820-4947

Email: [dharmendra30oct@gmail.com](mailto:dharmendra30oct@gmail.com)

**Dr. Mi-hyun Kim, Ph.D**

Assistant Professor

Office: +82-32-820-4947

Email: k[mh0515@gachon.ac.kr](mailto:mh0515@gachon.ac.kr)

$contributed equally

**CONTENTS**

**Table S1.** Force Field Parameters for ROS under investigation.

**Figure S1.** Evolution of area per lipid for native skin-lipid bilayer membrane (CER+CHO+FFA)

**Figure S2.** Values of order parameter (Sz) along the (a) sn-1 and sn-2 chains of CER; (b) FFA chain.

**Figure S3.** Distribution of ROS species a) H2O2-25 and b) H2O2-50 in skin-lipid bilayer membrane (CER-CHO-FFA).

**Figure S4.** Distribution of ROS species a) O2-25 and b) O2-50 in skin-lipid bilayer membrane (CER-CHO-FFA).

**Figure S5.** Bootstrapping standard deviation profile of different reactive oxygen species (ROS) across skin-lipid bilayer (CER-CHO-FFA) membrane.

**Table S1. Force Field Parameters for ROS under investigation.**

**Reactive Oygen Species: HOOH (Hydrogen Peroxide)**

[ moleculetype ]

; Name nrexcl

HOOH 3

[ atoms ]

; nr type resnr resid atom cgnr charge mass total_charge

1 OP 1 HOOH O1 1 -0.42 15.9994

2 H 1 HOOH H2 1 0.42 1.0080 ; 0.000

3 OP 1 HOOH O3 2 -0.42 15.9994

4 H 1 HOOH H4 2 0.42 1.0080 ; 0.000

[ bonds ]

; ai aj funct c0 c1

1 2 2 0.0981 2.3064e+07 ; adopted from Swart et al.

1 3 2 0.1443 6.4702e+06

3 4 2 0.0981 2.3064e+07

[ exclusions ]

; ai aj

2 4 ; atoms involved in a RB-type dihedral

[ angles ]

; ai aj ak funct angle fc

2 1 3 2 100.36 524.48 ; adopted from Swart et al.

1 3 4 2 100.36 524.48

[ dihedrals ]

; ai aj ak al funct c0 c1 c2 c3 c4 c5

2 1 3 4 3 1.99544235 -10.72695 15.22612 -2.24420 0.25839 0.00000 ; adapted from Koput et al.

**Reactive Oygen Species: HOO (Hydroperoxyl radical)**

[ moleculetype ]

; Name nrexcl

HOO 3

[ atoms ]

; nr type resnr resid atom cgnr charge mass total_charge

1 OP 1 HOO O1 1 -0.42 15.9994

2 H 1 HOO H2 1 0.494 1.0080

3 O 1 HOO O3 1 -0.074 15.9994 ; 0.000

[ bonds ]

; ai aj funct c0 c1

1 2 2 0.0981 2.3064e+07 ; adopted from Swart et al.

1 3 2 0.1443 6.4702e+06

[ angles ]

; ai aj ak funct angle fc

2 1 3 2 100.36 524.48 ; adopted from Swart et al.

**Reactive Oygen Species: HO (Hydroxyl radical)**

[ moleculetype ]

; Name nrexcl

HO 3

[ atoms ]

; nr type resnr resid atom cgnr charge mass total_charge

1 OP 1 HO O1 1 -0.436 15.9994

2 H 1 HO H2 1 0.436 1.0080 ; 0.000

[ bonds ]

; ai aj funct c0 c1

1 2 2 0.0981 2.3064e+07 ; adopted from Swart et al.

**Reactive Oxygen Species: O_2_ (molecular oxygen)**

[ moleculetype ]

; Name nrexcl

O2 3

[ atoms ]

; nr type resnr resid atom cgnr charge mass total_charge

1 OO 1 O2 O1 1 0 15.9994

2 OO 1 O2 O2 2 0 15.9994 ; 0.000

[ bonds ]

; ai aj funct c0 c1

1 2 2 0.1210 1.6600e+07 ; bond length from CRC handbook and constant from gb_5

**Figure S1.** Evolution of area per lipid for native skin-lipid bilayer membrane (CER+CHO+FFA)**.**

**
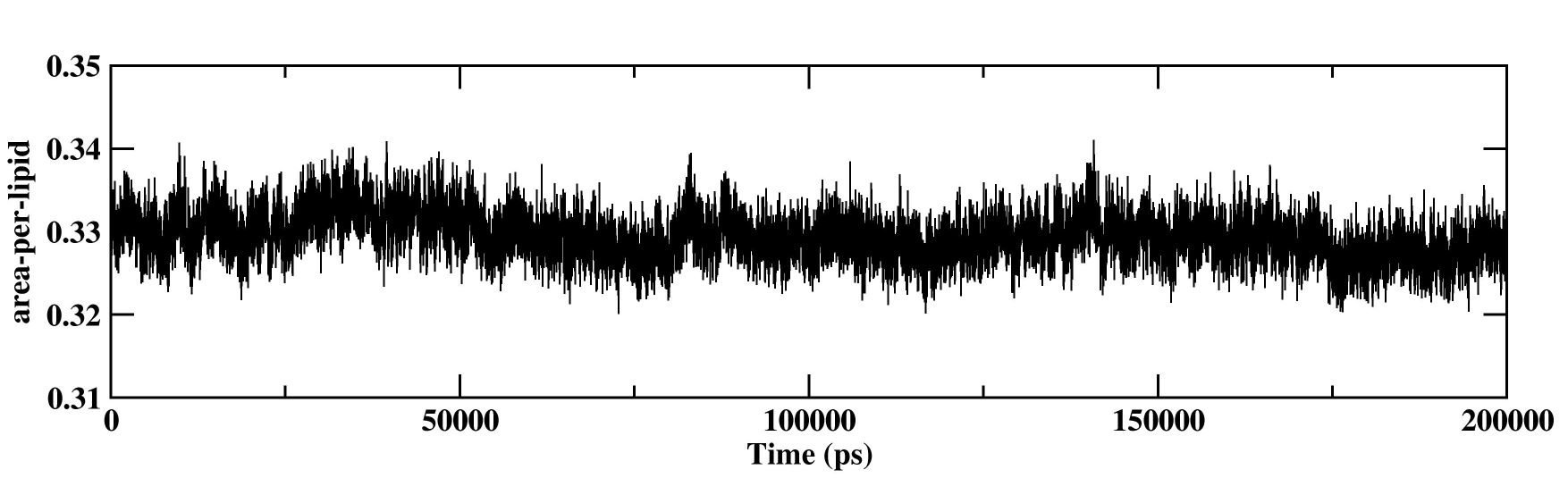
**

**Figure S2.** Values of order parameter (S_z_) along the **(a)** sn-1 and sn-2 chains of CER; **(b)** FFA chain.

**
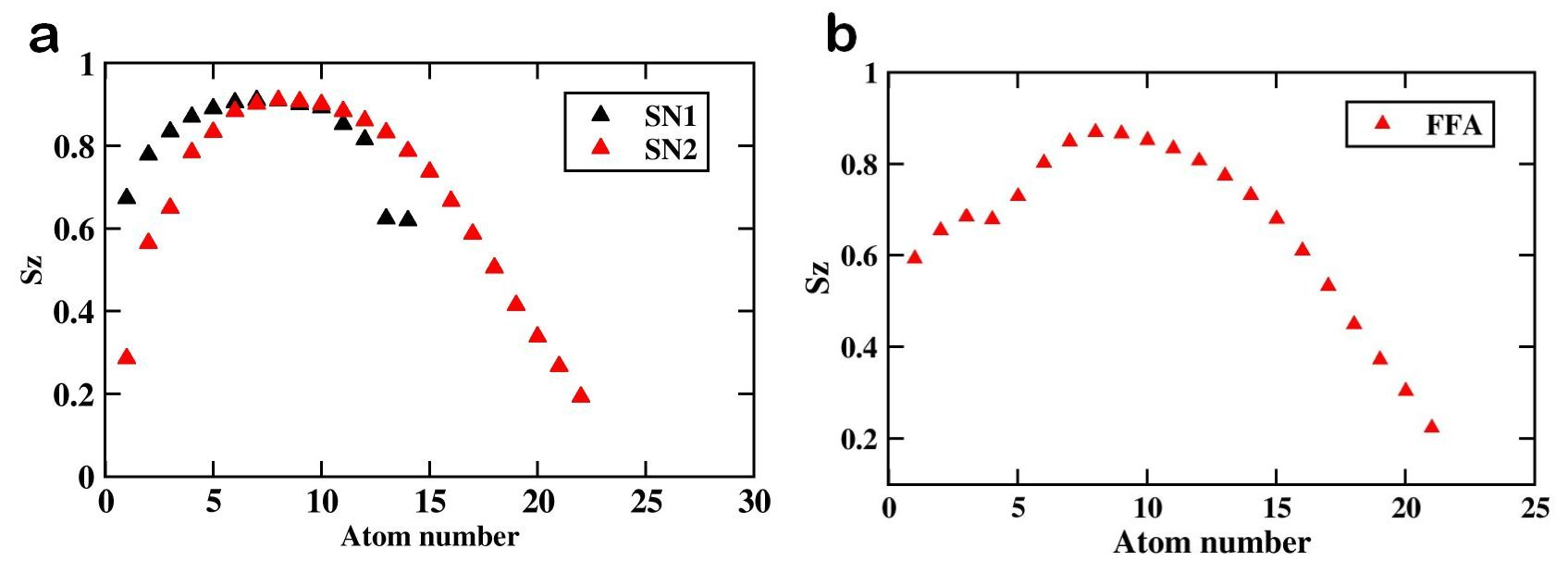
**

**Figure S3.** Distribution of ROS species a) H_2_O_2_-25 and b) H_2_O_2_-50 in skin-lipid bilayer membrane (CER-CHO-FFA).


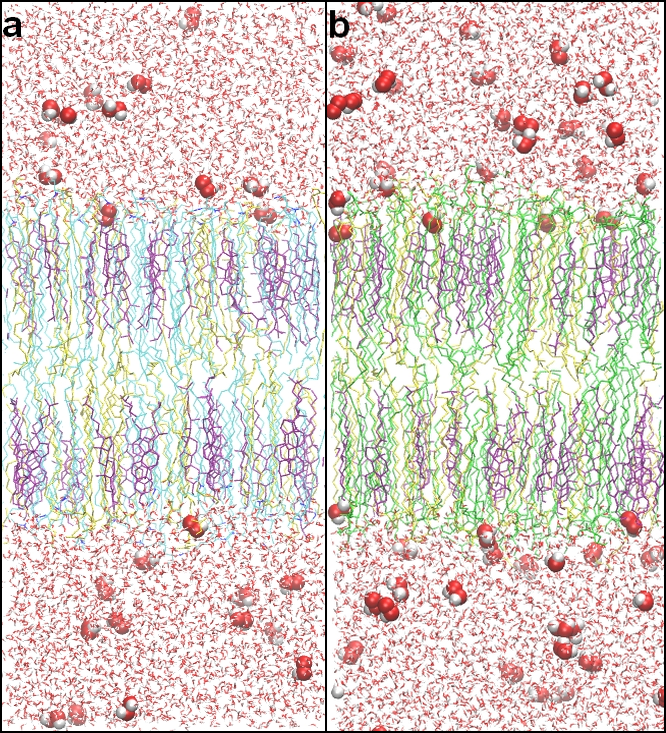


**Figure S4.** Distribution of ROS species a) O_2_-25 and b) O_2_-50 in skin-lipid bilayer membrane (CER-CHO-FFA).


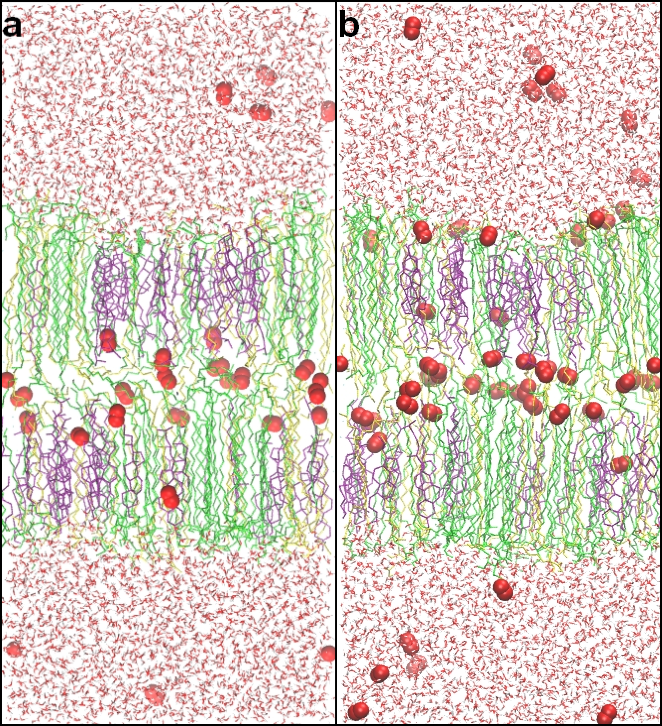


**Figure S5.** Bootstrapping standard deviation profile of different reactive oxygen species (ROS) across skin-lipid bilayer (CER-CHO-FFA) membrane.

**
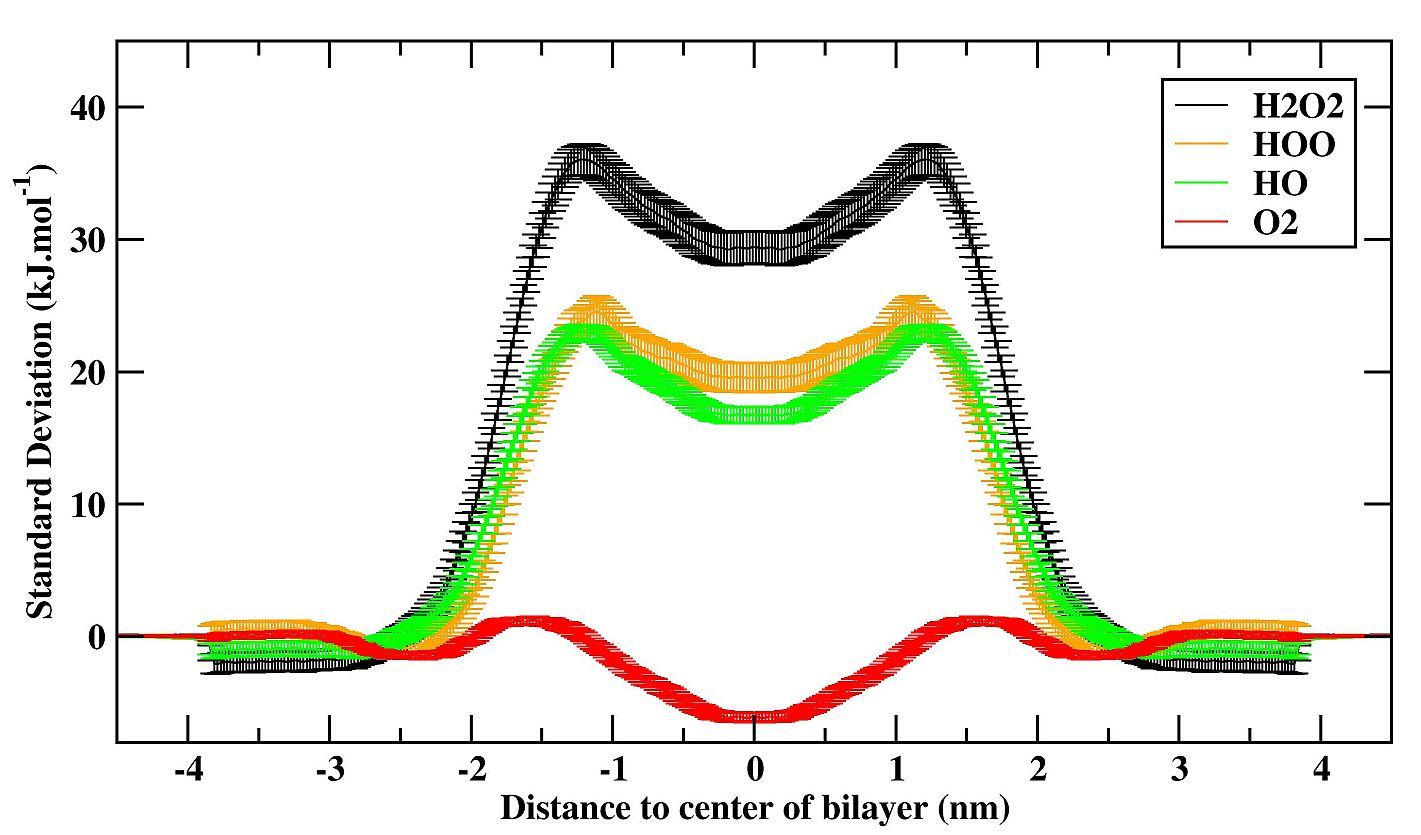
**

**REFERENCES**

Haynes, W.M. (2014). *CRC handbook of chemistry and physics.* CRC press.

Koput, J., Carter, S., and Handy, N.C. (1998). Potential Energy Surface and Vibrational− Rotational Energy Levels of Hydrogen Peroxide. *The Journal of Physical Chemistry A* 102(31)**,** 6325-6330.

Swart, M. (2002). *Density functional theory applied to copper proteins.* University Library Groningen][Host].
